# Diverse Radiologic Presentations of Paget’s Disease of the Breast: From Subtle to Striking Findings

**DOI:** 10.7759/cureus.114011

**Published:** 2026-08-05

**Authors:** Madhumita Sugumaran, Bhawna Dev, Archana Balasubramanian, Preetha Nethaji, Harini Gnanavel, Jai Prakash Srinivasan, Venkata Sai

**Affiliations:** 1 Radiology, Sri Ramachandra Institute of Higher Education and Research, Chennai, IND; 2 Pathology, Sri Ramachandra Institute of Higher Education and Research, Chennai, IND

**Keywords:** breast mri, ductal carcinoma in situ, mammography, multimodality imaging, nipple-areolar complex, paget’s disease of the breast

## Abstract

Objectives

Paget’s disease of the breast is a rare condition that is often associated with underlying malignancy. Given the subtle mammographic findings, a multimodal imaging approach is essential for an early diagnosis. The objective of this study was to describe the clinical, multimodality imaging, and histopathological spectrum of histopathologically confirmed mammary Paget's disease in eight patients.

Materials and methods

This is a descriptive study of eight histopathologically confirmed patients with Paget's disease of the breast, identified over a 10-year period (2015-2025) at a tertiary care center in India. Imaging modalities analyzed included mammography, ultrasonography, MRI, and positron emission tomography-computed tomography (PET-CT), with clinical and pathological correlation.

Results

The patients’ ages ranged from 27 to 75 years, with one case of bilateral disease. Nipple ulceration was the predominant symptom in six patients (75%), followed by breast lump in two patients (25%). Mammography was performed in five patients (63%) and showed irregular equal-density mass, round circumscribed nodule, fine pleomorphic calcifications, skin thickening, nipple retraction, and axillary lymphadenopathy; notably, all mammograms in this study had features suggestive of malignancy, in contrast to previous studies. Ultrasonography was performed in seven patients (88%); irregular hetero-echoic masses, thickened nipple-areola complexes with internal vascularity, ductal involvement, heterogeneous parenchyma with calcifications, and axillary lymphadenopathy were common. MRI was performed in three patients (38%) and showed irregular enhancing lesions, satellite nodules, ductal involvement, nonmass enhancement, and nipple erosion. PET-CT was performed in two patients (25%) and showed fluorodeoxyglucose (FDG)-avid ill-defined lesions with associated lymphadenopathy. One multifocal disease pattern was identified on MRI, and one multicentric disease pattern was identified on mammography. Surgical management included modified radical mastectomy in five patients (63%), simple mastectomy in two patients (25%), and wide local excision in one patient (12%). Histopathological analysis revealed Paget’s disease with underlying invasive mammary carcinoma in four patients (50%), ductal carcinoma in situ (DCIS) in two patients (25%), and isolated Paget’s disease with no underlying malignancy in two patients (25%).

Conclusion

Paget’s disease presents with subtle radiological features. Hence, a multimodality imaging strategy may improve characterization and assessment of disease extent and help in planning treatment.

## Introduction

Paget's disease of the breast is characterized by malignant glandular epithelial cells within the epidermis of the nipple-areolar complex, usually associated with underlying ductal carcinoma in situ (DCIS) or invasive carcinoma [1]. This condition constitutes 1%-3% of all breast cancer cases and predominantly affects postmenopausal women [2]. The age distribution ranges from 20 to 90 years, with the highest incidence observed in individuals in their 60s and 70s [3]. The disease is primarily unilateral, with bilateral involvement being rare [4]. It may be associated with DCIS and/or invasive carcinoma affecting the breast parenchyma [5]. Two principal theories elucidate the pathogenesis of Paget’s disease of the breast. The most widely endorsed is the epidermotropic theory, which proposes that the migration of Paget DCIS cells along the basement membrane of the nipple is facilitated by the motility factor heregulin α, acting through the HER2 receptor. This theory is corroborated by the presence of DCIS in the deeper breast tissue, which is identical to Paget cells in the majority of cases [6]. The transformation theory suggests that Paget cells originate from the malignant transformation of keratinocytes or Toker cells; this theory accounts for rare cases (< 5%) of Paget’s disease where there is no underlying breast malignancy [7]. Most patients experience a gradual onset of the disease and exhibit clinical symptoms such as changes in the nipple, including erythema, crusting, ulceration, excoriation, an eczematoid rash, itching, or bleeding [5]. Approximately 50% of patients present with a palpable mass, highly indicative of underlying invasive carcinoma [8]. In cases where symptoms are present, the diagnosis is primarily determined through clinical assessment and subsequently verified by histopathological analysis. The skin punch biopsy is the most commonly used procedure, whereas the core needle biopsy is less frequently employed [9]. Imaging plays a pivotal role in the detection and assessment of the extent of underlying malignancies, thereby facilitating appropriate treatment planning. Mammography is capable of identifying malignant masses, architectural distortions, or calcifications [10]; however, it is noteworthy that approximately half of the patients with mammary Paget’s disease present with normal mammograms [11,12]. Ultrasound may reveal duct ectasia, underlying masses, or alterations in the nipple-areolar complex, such as nipple retraction, asymmetry, and skin thickening [13]. Magnetic resonance imaging is particularly sensitive in identifying mammographically occult underlying disease [14]. The prognosis is predominantly influenced by the presence, extent, and characteristics of the underlying tumor. Patients diagnosed with noninvasive Paget’s disease of the nipple generally experience favorable outcomes [5]. Conversely, individuals with Paget's disease accompanied by DCIS exhibit poorer survival rates compared to those with isolated Paget's disease of the nipple [15]. If an underlying invasive carcinoma is present, the prognosis is contingent upon tumor size and lymph node status, with the five-year relative survival rate decreasing as the tumor stage advances [3]. The selection of surgical intervention is influenced by the presence or absence of underlying breast carcinoma. For patients diagnosed with Paget’s disease-DCIS or invasive ductal carcinoma, breast-conserving therapy, which includes lumpectomy and radiation, is an oncologically equivalent local treatment option to mastectomy in appropriately selected patients with negative margins and limited disease extent, as supported by current surgical oncology evidence [16,17]. Given that mammographic findings may be absent or nonspecific in a significant proportion of cases, a structured multimodality imaging approach incorporating ultrasonography, MRI, and PET-CT is important for disease detection and extent assessment. The objective of this study was to describe the multimodality imaging spectrum of histopathologically confirmed mammary Paget's disease in a retrospective case series of eight patients.

## Materials and methods

**Figure 1:**
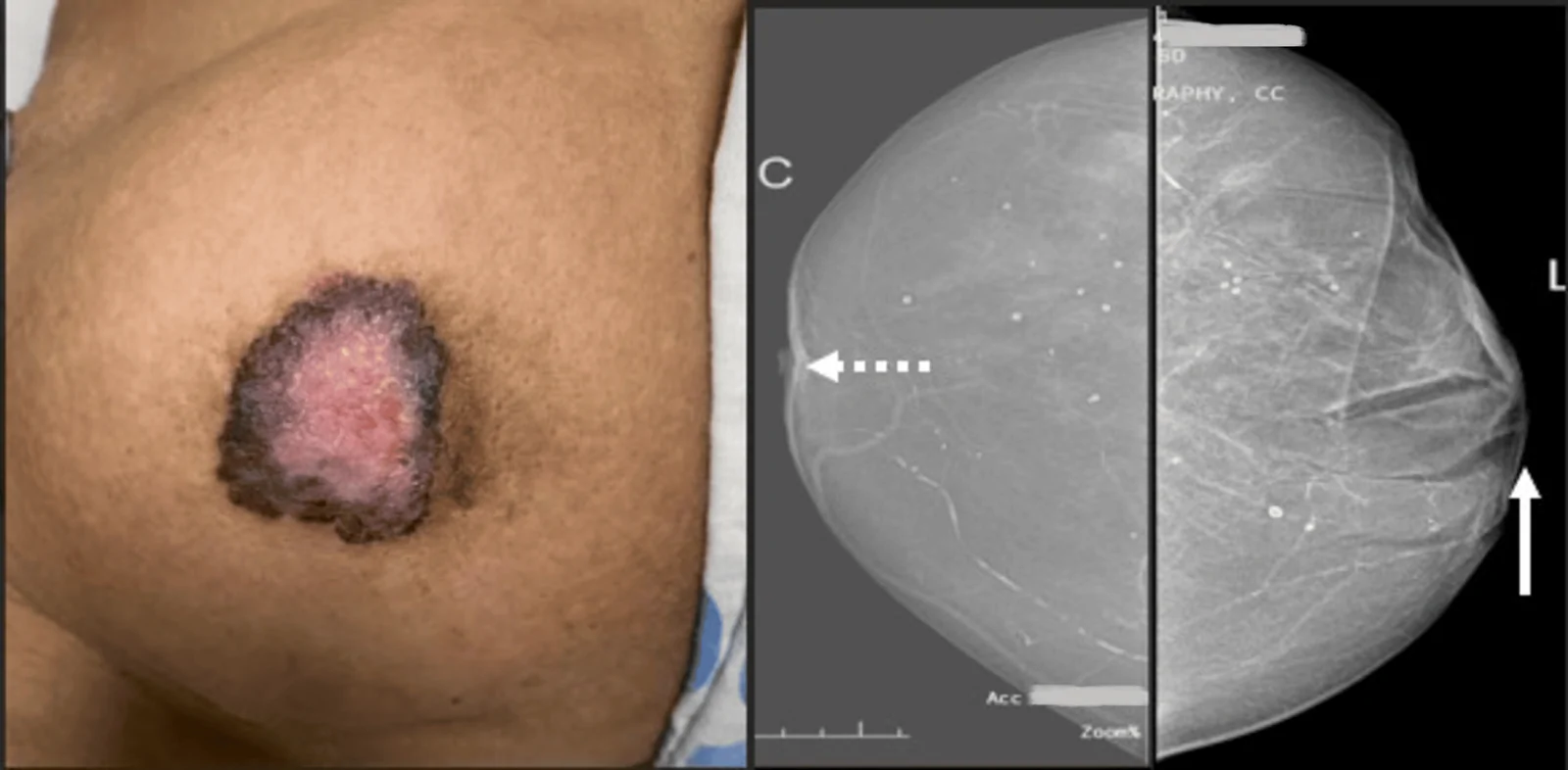
Clinical picture and mammogram of patient with Paget’s disease of the left nipple CC: craniocaudal (mammographic view) Clinical picture of nipple destruction and excoriation, proven Paget’s disease of the left nipple on punch biopsy. Mammogram showing a normal nipple (dotted arrow) on the right breast CC view and no nipple (arrow) seen on the left breast CC view

This descriptive study was conducted at a tertiary care hospital in India from 2015 to 2025. The study was approved by the Institutional Ethics Committee of Sri Ramachandra Institute of Higher Education and Research (CSP-MED/25/DEC/123/301). Patients with histopathologically confirmed Paget's disease of the breast who underwent imaging and had complete clinical and surgical records were identified through the institutional radiology and pathology databases and included in the study. Clinical examination findings, including characteristic nipple-areolar changes (Figure 1), were documented from the medical records. Patients without a definitive histopathological diagnosis, those with incomplete data, and those who had undergone prior interventions affecting imaging interpretation were excluded.

Imaging findings were described using the American College of Radiology (ACR) Breast Imaging Reporting and Data System (BI-RADS) lexicon terminology, with a BI-RADS category assigned to each examination. Modality selection was clinically driven; modality-specific percentages were calculated using the number of patients who underwent that modality as the denominator. Data are reported as frequencies and percentages. Images were reviewed by two radiologists with experience in breast imaging, with consensus used to resolve disagreements.

Mammography was performed in standard craniocaudal (CC) and mediolateral oblique (MLO) projections, with spot magnification views where indicated. Ultrasonography was performed using the APLIO 400 and SAMSUNG V8 systems with a 4-18 MHz linear transducer incorporating color Doppler evaluation. MRI was performed on a 1.5T system using a dedicated breast coil, including T2-weighted fat-suppressed, diffusion-weighted, and dynamic contrast-enhanced T1-weighted sequences following intravenous gadolinium administration. Positron emission tomography-computed tomography (PET-CT) was performed 60 minutes after intravenous fluorine-18 fluorodeoxyglucose (18F-FDG) administration, with SUVmax recorded for all FDG-avid lesions.

## Results

Eight cases of Paget's disease of the breast with imaging studies were identified over 10 years. The mean age was 57.5 years, and the median age was 62.5 years. The majority of the study population belonged to the age group of 40 to 75 years, while one patient was diagnosed below 30 years of age with bilateral Paget's disease of the breast. The most common clinical presentation was nipple ulceration in six patients (75%), followed by breast lump in two patients (25%) (Table 1).

**Table 1:** Summary of age, clinical presentation, imaging modalities performed and its principal findings, histopathological diagnosis, and surgical management of all eight patients (cases 1-8) USG: ultrasonography; PET-CT: positron emission tomography-computed tomography; NAC: nipple-areolar complex; LOQ: lower outer quadrant; LIQ: lower inner quadrant; MRM: modified radical mastectomy; UOQ: upper outer quadrant; FDG: fluorodeoxyglucose; DCIS: ductal carcinoma in situ

Case	Age/sex	Clinical presentation	Modalities performed	Principal findings	Lesion location	Histopathology	Surgery
1	72F	Right breast lump	Mammography, USG	Nipple retraction; round, circumscribed, equal-density nodule in both breasts	Right breast	Paget's + DCIS	MRM
2	59F	Right nipple ulceration + bleeding	USG, PET-CT	Irregular skin thickening/erosion of NAC, ↑vascularity, axillary lymphadenopathy; FDG-avid nodular skin thickening	Right NAC	Paget's + invasive mammary carcinoma	MRM + axillary clearance
3	66F	Left nipple ulceration	MRI (USG used for biopsy guidance only)	Nipple erosion, skin ulceration, subareolar thickening; enhancing circumscribed mass with diffusion restriction/ADC drop	Left nipple/subareolar region	Paget's + invasive ductal carcinoma	MRM
4	27F	Bilateral nipple ulceration	USG, MRI	Bilateral NAC skin thickening, hypoechoic nonmass area with calcific specks, bilateral axillary lymphadenopathy; nonmass enhancement LOQ left, intense NAC enhancement right	Bilateral (LOQ left; NAC right)	Paget's (bilateral), no malignancy	Bilateral WLE
5	71F	Left breast lump	Mammography, USG, MRI, PET-CT	Irregular equal-density mass with pleomorphic calcifications; hypoechoic mass with vascularity; enhancing mass with ductal enhancement; FDG-avid lesion	LIQ/subareolar, left breast	Paget's + invasive mammary carcinoma	MRM
6	75F	Left nipple ulceration	Mammography, USG	Enlarged left axillary node with retroareolar skin thickening; cortical thickening of node	Left retroareolar region/axilla	Isolated Paget's, no malignancy	Simple mastectomy
7	43F	Left nipple ulceration	Mammography, USG	Fine linear pleomorphic calcifications, segmental/regional; hetero-echoic NAC with calcifications	Left breast (retroareolar)	Paget's + DCIS	Simple mastectomy
8	47F	Right nipple ulceration	Mammography, USG	Retroareolar skin thickening; calcifications in two separate quadrants (multicentric)	Right breast (LIQ + UOQ)	Paget's + invasive mammary carcinoma	MRM + axillary clearance

Mammogram

Mammography was performed in five patients (63%). The findings noted were an enlarged axillary lymph node with skin thickening in one patient (20%), an irregular equal-density mass with nipple retraction and regional fine pleomorphic calcifications in one patient (20%), a round circumscribed equal-density nodule with nipple retraction in one patient (20%), grouped fine pleomorphic calcification in one patient (20%), and fine linear pleomorphic calcifications in segmental and regional distribution in one patient (20%).

Ultrasonogram

Ultrasonography was performed in seven patients (88%). The findings noted were axillary lymphadenopathy with thickened cortex in one patient (14%), irregular heterogeneously hypoechoic masses with microlobulated margins, calcification, and posterior acoustic shadowing in one patient (14%), heterogeneous parenchyma with abnormally dilated ducts showing calcifications extending from the nipple in one patient (14%), an irregular predominantly hypoechoic mass with internal vascularity and nipple retraction in one patient (14%), markedly irregular skin thickening with increased vascularity and erosion of the nipple-areola complex in one patient (14%), hetero-echoic nipple-areolar complex and subareolar region with calcifications and increased vascularity in one patient (14%), and an irregular predominantly hypoechoic nonmass area with calcific specks and bilateral skin thickening involving the nipple-areolar complex with associated lymphadenopathy in one patient (14%).

Magnetic resonance imaging

Magnetic resonance imaging was performed in three patients (38%) (Table 1). The findings noted were an irregular circumscribed mass with satellite foci associated with nonmass linear ductal enhancement and abnormal asymmetric nipple enhancement in one patient (33%), nipple erosion with skin ulceration, subcutaneous edema, focal enhancing subareolar thickening and adjacent linear enhancement, and an enhancing circumscribed round mass showing diffusion restriction with associated axillary lymphadenopathy in one patient (33%), and a case of bilateral Paget’s disease of the breast with intensely enhancing nipple-areolar region of the right breast and an irregular nonmasslike area in the left breast with involvement of the nipple-areolar complex in one patient (33%).

PET

PET was performed in two patients (25%). The findings noted were ill-defined FDG-avid lesions with skin thickening and associated lymphadenopathy in one patient (50%) and an ill-defined large FDG-avid-enhancing nodular skin thickening with associated lymphadenopathy in one patient (50%).

There was one multifocal presentation, identified on MRI, and one multicentric presentation, identified on mammography, in the study population. The majority of patients, five (63%), underwent modified radical mastectomy, followed by simple mastectomy in two patients (25%) and wide local excision in one patient (12.5%) with less extensive involvement. The most common pathological type was Paget’s disease of the breast with invasive mammary carcinoma in four patients (50%), followed by DCIS in two patients (25%) and Paget’s disease of the nipple without underlying breast carcinoma in two patients (25%).

Case 1

**Figure 2:**
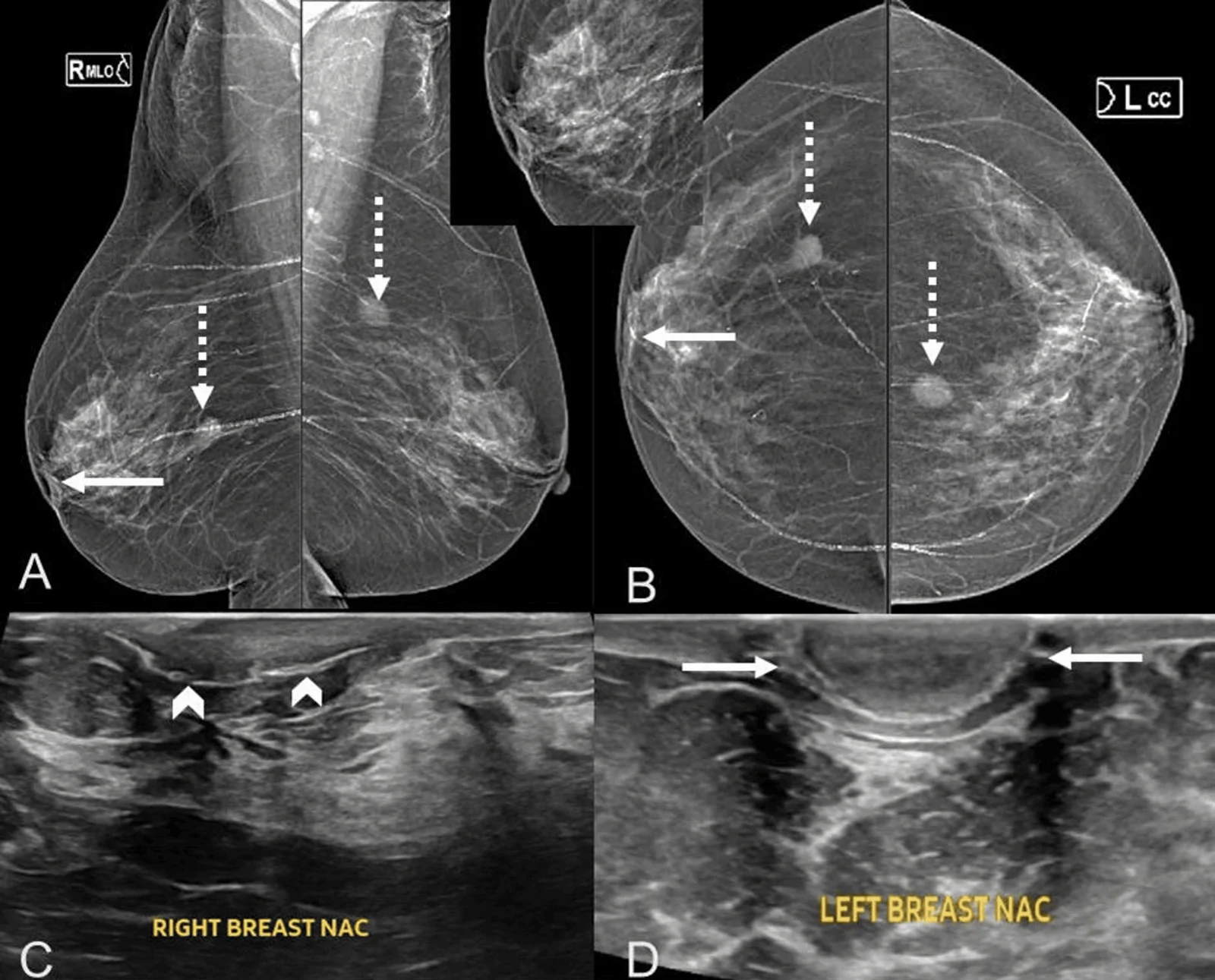
Mammographic and ultrasonographic findings demonstrating right nipple retraction with comparison to the normal contralateral nipple-areola complex (A and B) Mammogram showing retraction of the right nipple (solid white arrow), also showing a round, circumscribed, equal-density nodule in each breast (dotted white arrow). (C) Ultrasonogram representing retraction of the right nipple (arrowhead). (D) Normal left nipple-areolar complex (solid white arrows)

**Figure 3:**
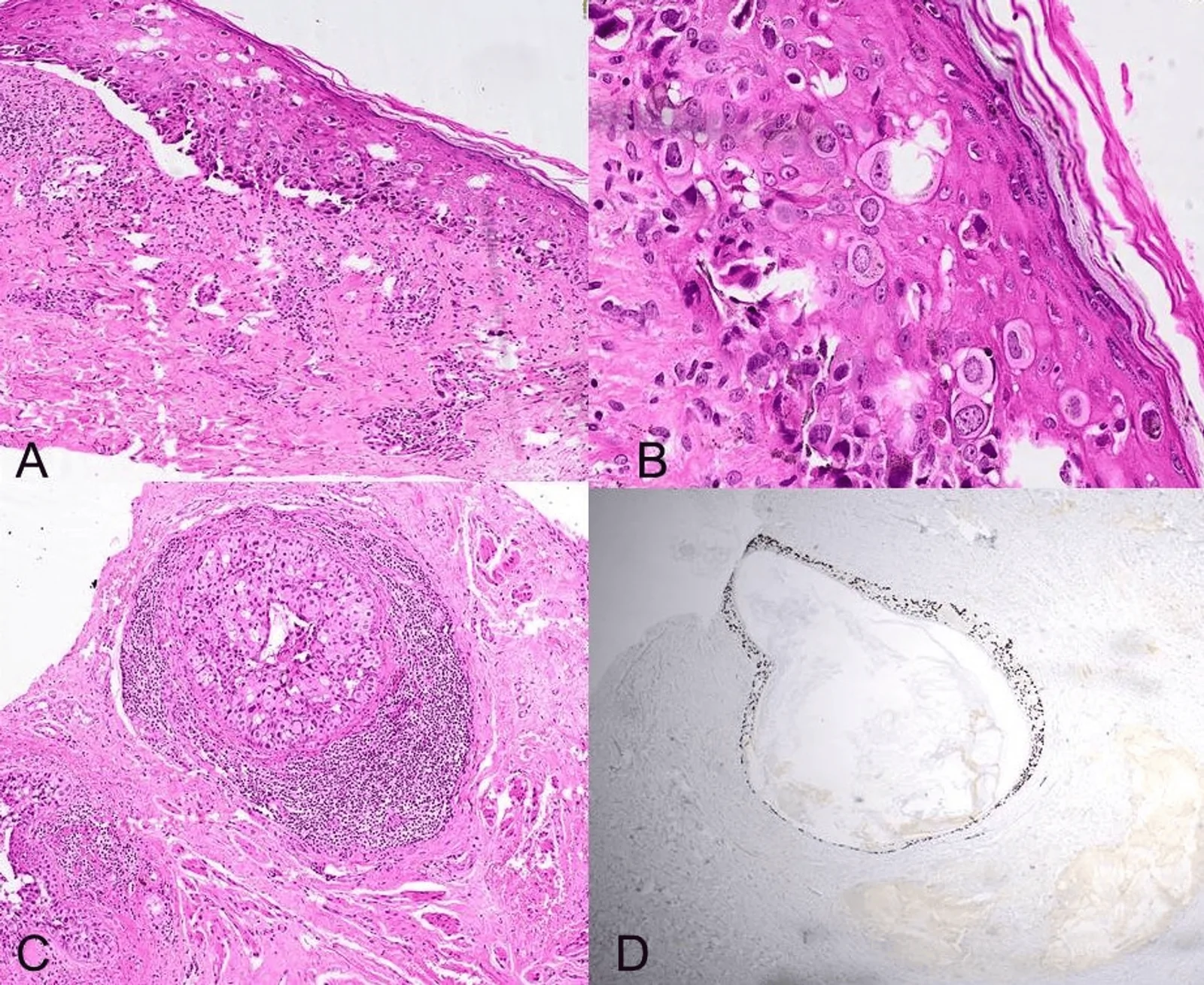
Histopathological and immunohistochemical findings demonstrating intraepithelial atypical cell proliferation and DCIS DCIS: ductal carcinoma in situ; H&E: hematoxylin and eosin; IHC: immunohistochemistry (A and B) H&E, 10X. Photomicrograph of edge wedge biopsy from a nipple-areola complex shows stratified squamous epithelium with intraepithelial proliferation of atypical cells. (C) H&E, 40X. Photomicrograph of DCIS component showing a dilated duct filled with large atypical cells surrounded by dense lymphocytic infiltrate. (D) IHC, 40X. P63 highlights the myoepithelial cells in the periphery of the dilated duct, confirming DCIS

A 72-year-old female patient presented with a right breast lump of six months’ duration, with no associated nipple discharge, ulceration, or pain. On clinical examination, a firm, nontender lump was palpable in the right breast, and minimal retraction of the right nipple was noted, while the left nipple-areola complex appeared clinically normal. Mammography demonstrated retraction of the right nipple along with an equal-density nodule in both breasts (Figures 2a-2b). Ultrasonography confirmed retraction of the right nipple and a normal-appearing left nipple-areola complex (Figures 2c-2d). Punch biopsy of the right nipple-areola complex showed stratified squamous epithelium with intraepithelial proliferation of large atypical cells, confirming Paget’s disease, with an underlying DCIS component on histopathology, which was further confirmed by P63 immunohistochemistry highlighting the myoepithelial layer surrounding the involved ducts (Figure 3). The patient subsequently underwent modified radical mastectomy. As the disease was localized to an in situ component without evidence of nodal or distant spread, a favorable long-term outcome was anticipated; postoperative recovery was uneventful, and the patient remained disease-free on follow-up, consistent with this expectation.

Case 2

**Figure 4:**
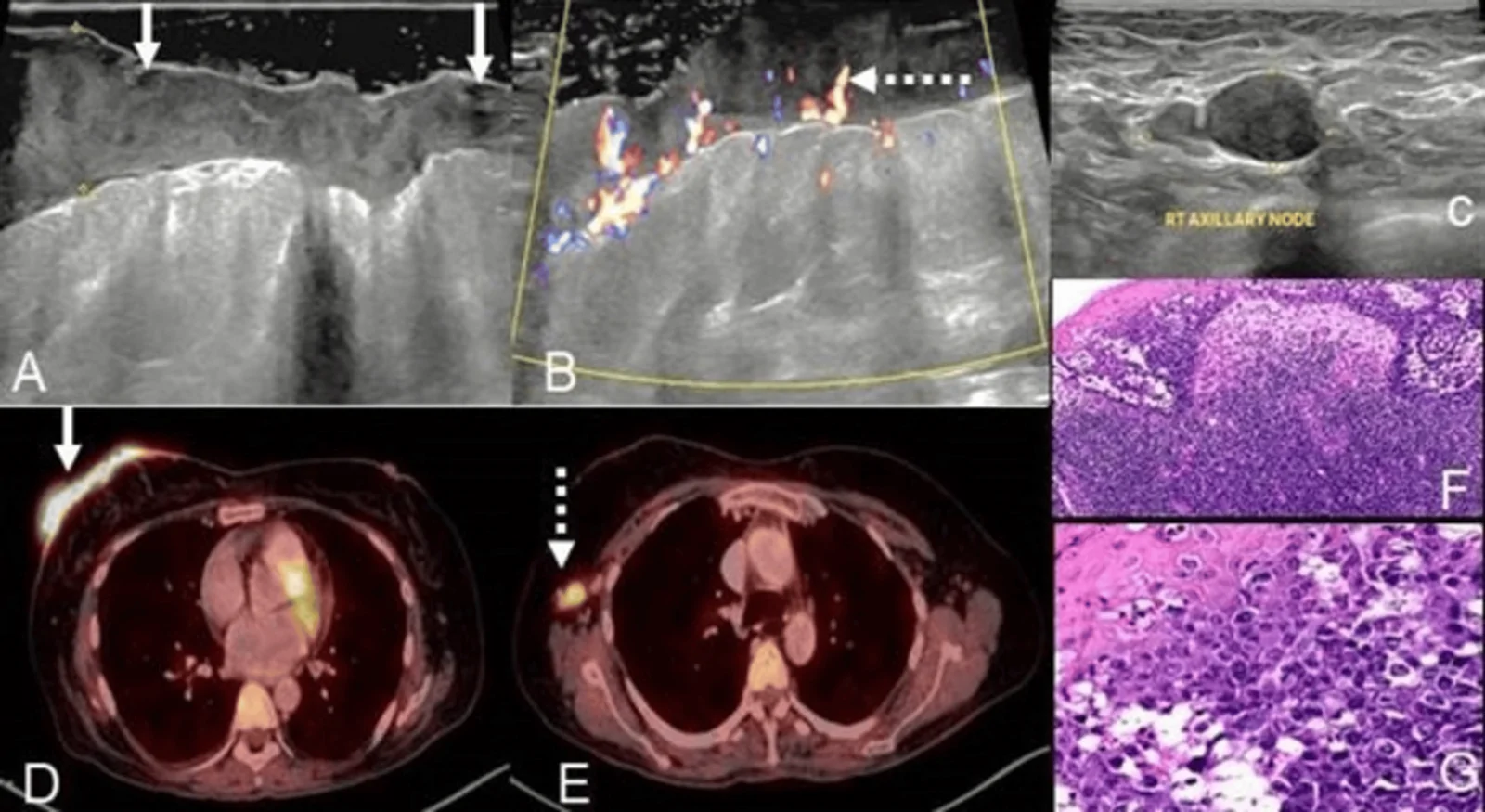
Multimodality imaging and histopathological findings demonstrating an ill-defined, large, FDG-avid, enhancing nodular skin thickening with associated axillary lymphadenopathy H&E: hematoxylin and eosin; PET: positron emission tomography; FDG: fluorodeoxyglucose (A-C) Ultrasonogram illustrating markedly irregular skin thickening, erosion of the nipple-areola complex (solid arrows) with increased vascularity (dotted arrow), and associated axillary lymphadenopathy. (D-E) PET demonstrating ill-defined large FDG-avid enhancing nodular skin thickening (solid arrow) and axillary lymphadenopathy (dotted arrow). (F) H&E, 10X. Photomicrograph of edge wedge biopsy from breast ulcer shows epidermis with a single atypical cell and clusters of large atypical cells. Underlying dermis shows extensive chronic inflammatory infiltrate. (G) H&E, 40X. Photomicrograph shows single cells and clusters of large atypical cells infiltrating the epidermis with prominent nucleoli. Underlying dermis also shows infiltrating malignant cells with nuclear pleomorphism and atypical mitotic figures

A 59-year-old female patient presented with right nipple ulceration and bleeding of six months’ duration. On examination, there was markedly irregular skin thickening with erosion of the nipple-areola complex and visibly increased vascularity, along with a palpable right axillary lymph node. Ultrasonography confirmed the markedly irregular skin thickening with erosion of the nipple-areola complex, increased vascularity, and axillary lymphadenopathy. PET-CT demonstrated an ill-defined, large, FDG-avid, enhancing nodular skin thickening with associated axillary lymphadenopathy, suggestive of locally advanced disease (Figure 4). Punch biopsy from the breast ulcer showed epidermis infiltrated by single cells and clusters of large atypical cells with extensive chronic inflammatory infiltrate in the underlying dermis; higher magnification confirmed infiltrating malignant cells with marked nuclear pleomorphism and atypical mitotic figures, consistent with Paget’s disease with an underlying invasive mammary carcinoma. The patient underwent modified radical mastectomy with axillary clearance. Given the locally advanced presentation with axillary nodal involvement, the prognosis was guarded and adjuvant therapy was planned following surgery; the patient tolerated the procedure well, with an uneventful postoperative course in keeping with the anticipated surgical outcome.

Case 3

**Figure 5:**
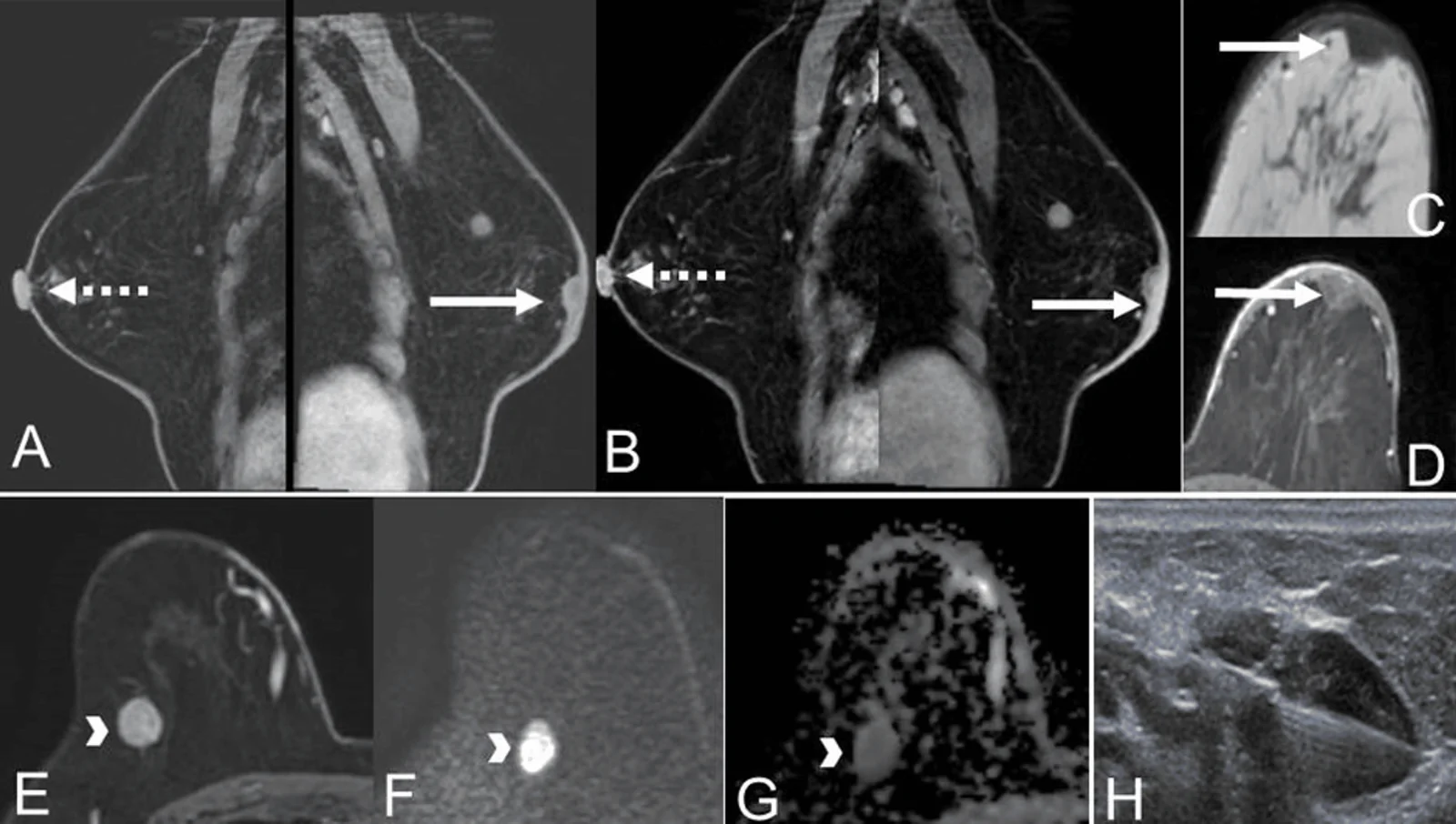
MRI and ultrasound-guided biopsy demonstrating left nipple involvement with an enhancing subareolar mass showing diffusion restriction ADC: apparent diffusion coefficient (A-D) MRI depicting nipple erosion, skin ulceration, subcutaneous edema, focal enhancing subareolar thickening of the left nipple (solid arrow), and the right normal nipple represented by dotted arrows. (E-G) Enhancing circumscribed round mass showing diffusion restriction and ADC signal drop (arrowhead). (H) Ultrasound-guided core needle biopsy from the mass

A 66-year-old female patient presented with left nipple ulceration of one year’s duration, without a palpable lump or nipple discharge. On examination, the left nipple showed surface erosion with adjacent skin ulceration, while the right nipple-areola complex was normal. MRI of the breast revealed nipple erosion, skin ulceration, and subcutaneous edema with focal enhancing subareolar thickening of the left nipple, while the right nipple appeared normal; additionally, an enhancing circumscribed round mass showing diffusion restriction with corresponding ADC signal drop was identified, from which an ultrasound-guided core needle biopsy was obtained (Figure 5). Histopathological examination confirmed Paget’s disease with an underlying invasive ductal carcinoma component. The patient subsequently underwent modified radical mastectomy. The patient had an uneventful postoperative recovery, consistent with this expectation. 

Case 4

**Figure 6:**
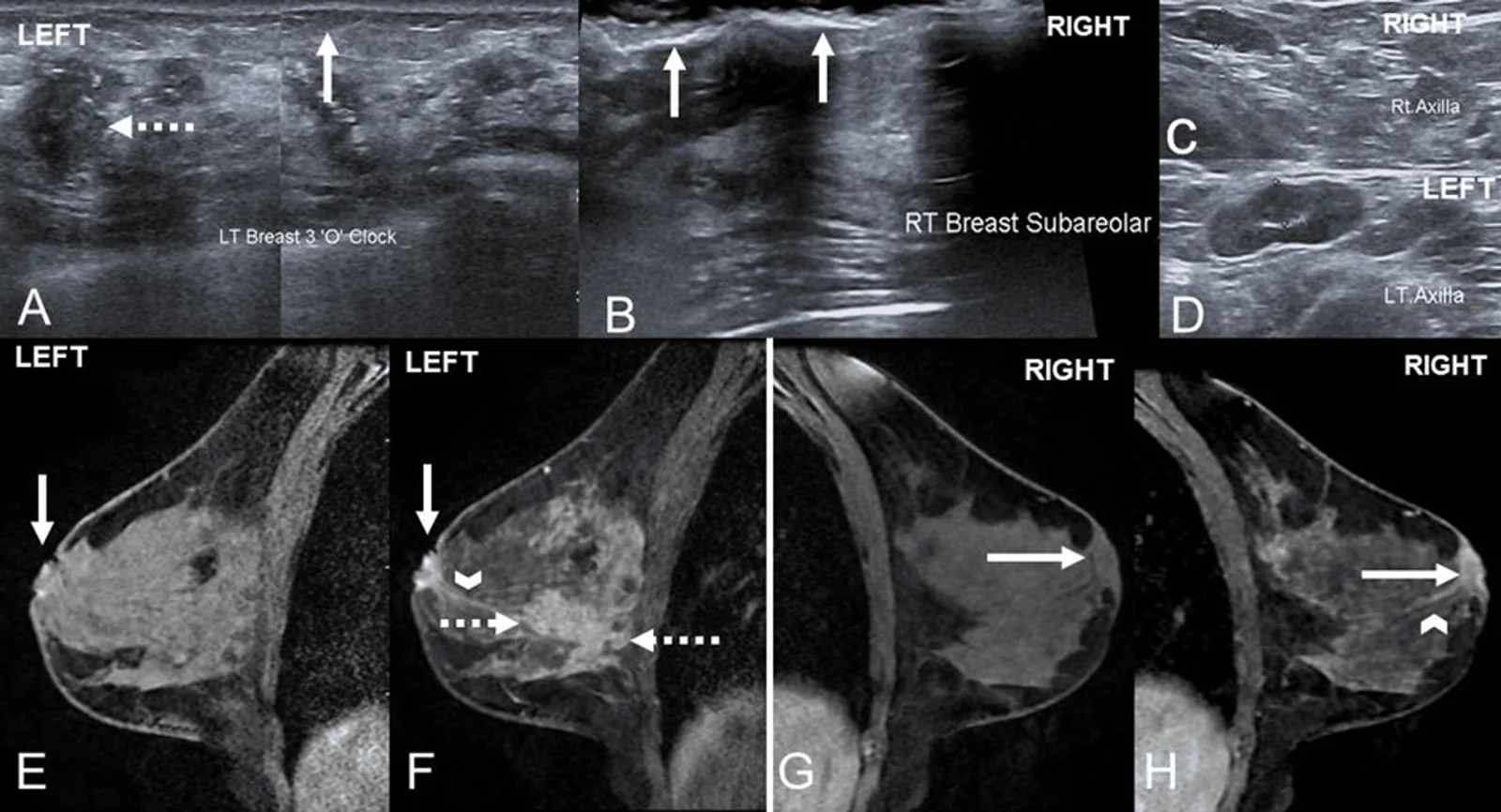
A case of bilateral Paget's disease of the breast CEMR: contrast-enhanced magnetic resonance (imaging) (A-B) Ultrasonogram demonstrating bilateral skin thickening involving the nipple-areola complex (solid arrows), irregular hypoechoic nonmass area with calcific specks (dotted arrow), and (C-D) bilateral axillary lymphadenopathy. (E-F) CEMR demonstrates nipple erosion (solid arrow), regional nonmass enhancement (dotted arrows) in the lower outer quadrant of the left breast extending to the eroded nipple (arrowhead). (G-H) Intensely enhancing nipple areolar complex (solid arrow), along with linear nonmass enhancement, right breast (arrowhead)

A 27-year-old female patient presented with bilateral nipple ulceration of two months’ duration, without a palpable lump in either breast. This was the youngest patient in the series and the only case of bilateral involvement. On examination, both nipple-areola complexes showed surface excoriation with skin thickening, and bilateral axillary lymph nodes were palpable. Ultrasonography demonstrated bilateral skin thickening involving the nipple-areola complex, an irregular hypoechoic nonmass area with calcific specks, and bilateral axillary lymphadenopathy. Contrast-enhanced MRI demonstrated nipple erosion with regional nonmass enhancement in the lower outer quadrant of the left breast extending to the eroded nipple, an intensely enhancing nipple-areolar complex on the right, and linear nonmass enhancement in the right breast (Figure 6). Bilateral punch biopsies confirmed Paget’s disease of both nipples. The patient underwent bilateral wide local excision with adjuvant therapy, given the bilateral and relatively limited extent of disease. As no underlying malignancy was identified, a good long-term outcome was anticipated despite bilateral involvement; the patient recovered well postoperatively and remained disease-free at follow-up, consistent with the expected favorable course. 

Case 5

**Figure 7:**
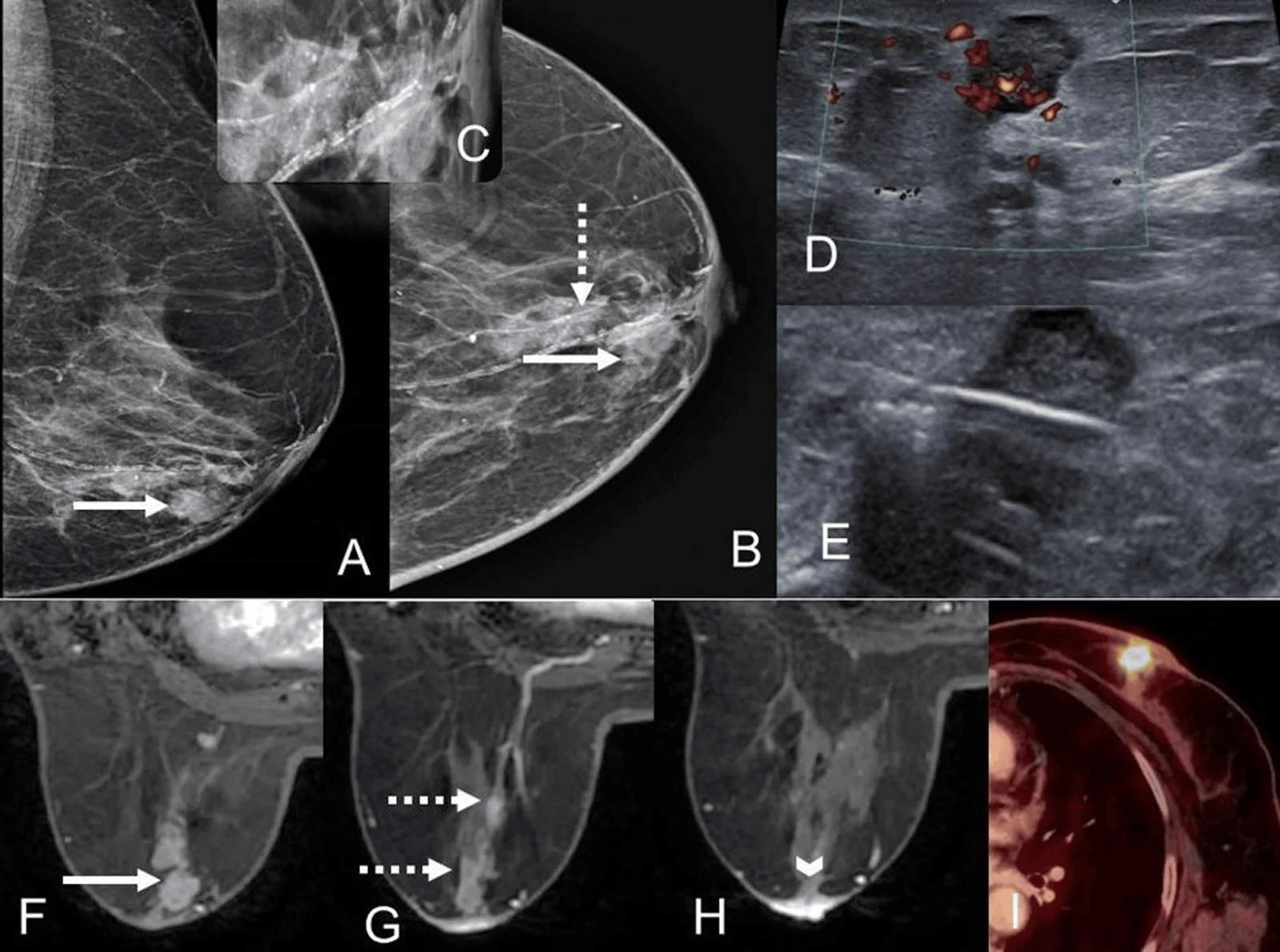
Multimodality imaging findings of left breast malignancy demonstrating an irregular mass with pleomorphic calcifications, ductal extension, nipple involvement, and FDG avidity PET: positron emission tomography; USG: ultrasonography; DCE MRI: dynamic contrast-enhanced magnetic resonance imaging; FDG: fluorodeoxyglucose (A and B) Mammogram demonstrating irregular equal-density masses (solid arrow) with regional fine pleomorphic calcifications (dotted white arrow) in the lower inner quadrant. (C) Zoomed view showing pleomorphic calcifications. (D) Ultrasonogram showing irregular heterogeneously hypoechoic masses with microlobulated margins with internal vascularity in the subareolar region of the left breast (solid arrow). (E) USG-guided core needle biopsy from the mass. (F-H) DCE MRI showing an enhancing irregular circumscribed mass (solid arrow) associated with nonmass linear ductal enhancement (dotted arrows) and abnormal nipple enhancement (arrowhead). (I) PET depicting an FDG-avid lesion with skin thickening

A 71-year-old female patient presented with a left breast lump of three months’ duration. On examination, an irregular, firm mass was palpable in the lower inner quadrant of the left breast. Mammography demonstrated irregular, equal-density masses with regional fine pleomorphic calcifications in the lower inner quadrant; magnified views confirmed the pleomorphic morphology of the calcifications. Ultrasonography showed irregular, heterogeneously hypoechoic masses with microlobulated margins and internal vascularity in the subareolar region of the left breast, from which an ultrasound-guided core-needle biopsy was obtained. MRI demonstrated an enhancing irregular circumscribed mass associated with nonmass linear ductal enhancement and abnormal nipple enhancement, while PET-CT depicted an FDG-avid lesion with associated skin thickening (Figure 7). Histopathological analysis of the core biopsy confirmed Paget’s disease with an underlying invasive mammary carcinoma. The patient underwent modified radical mastectomy. Given the presence of invasive carcinoma with satellite foci, adjuvant therapy was planned following surgery; postoperative recovery was uneventful, consistent with the anticipated outcome of locoregional control. 

Case 6

**Figure 8:**
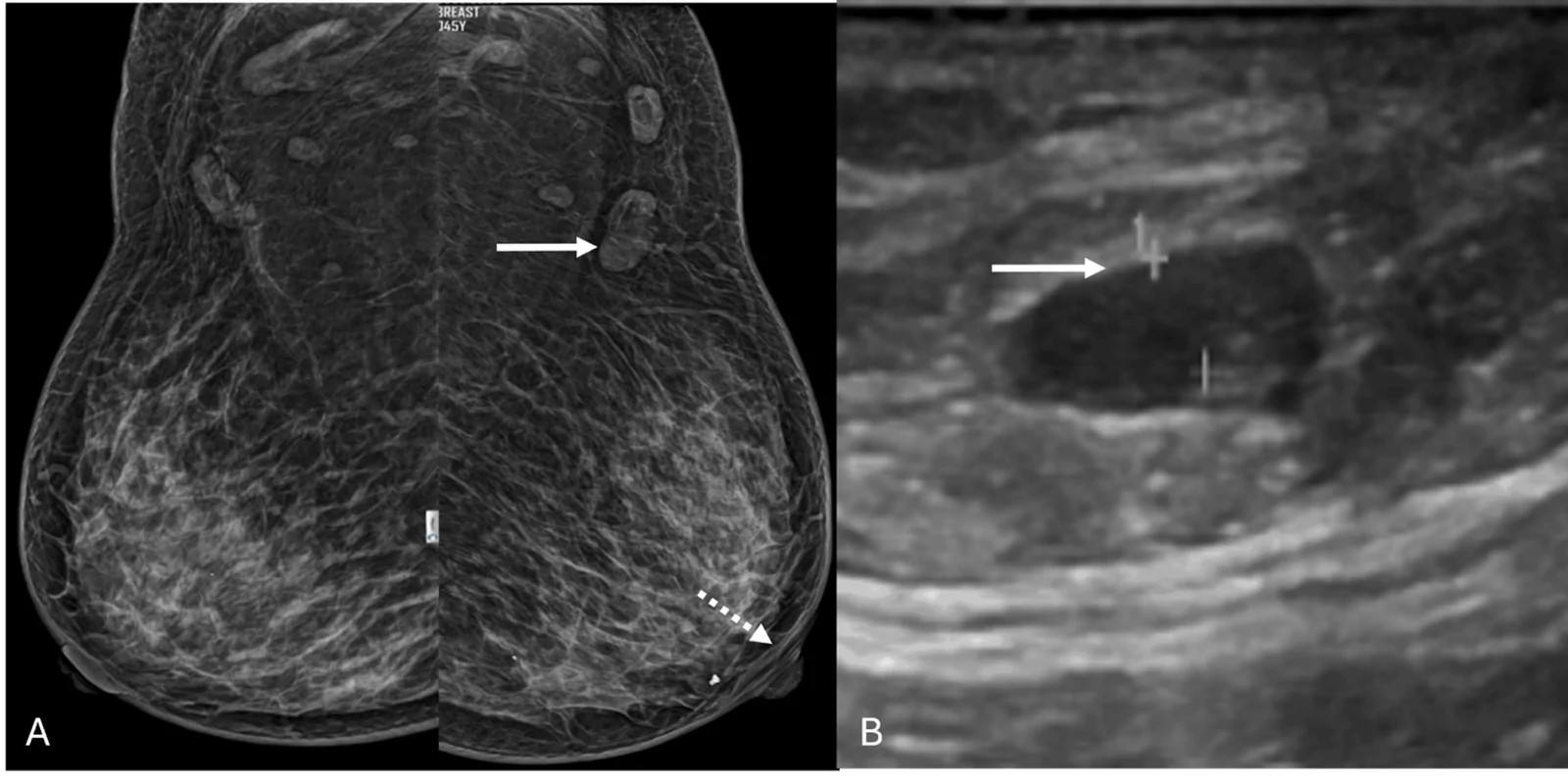
Mammographic and ultrasonographic findings demonstrating retroareolar skin thickening and abnormal left axillary lymphadenopathy MLO: mediolateral oblique (mammographic view) (A) Mammogram-MLO view of both breasts showing an enlarged left axillary lymph node (solid arrow) with skin thickening in the retroareolar region as compared to the right side (dotted arrow). (B) Ultrasonogram showing a level 1 left axillary abnormal lymph node with nonuniformly thickened cortex (solid arrow)

A 75-year-old female patient presented with ulceration of the left nipple of four months’ duration, without an associated palpable breast lump or nipple discharge. She had initially been treated empirically for presumed eczema at a local clinic, without symptomatic improvement, prompting referral for further evaluation. On examination, the left nipple showed surface erosion with overlying skin thickening in the retroareolar region, while no discrete lump was palpable in either breast; a palpable, nontender left axillary lymph node was noted, and the right breast and axilla were clinically unremarkable. Mammography revealed an enlarged left axillary lymph node with skin thickening in the retroareolar region, in contrast to the normal appearance of the right side. Ultrasonography confirmed a level I left axillary lymph node with nonuniform cortical thickening (Figure 8). Punch biopsy of the left nipple-areola complex demonstrated Paget cells confined to the epidermis, with no evidence of an underlying DCIS or invasive component on histopathology; the axillary lymph node enlargement was attributed to a reactive process secondary to the chronic nipple ulceration rather than nodal metastasis. Given the absence of underlying malignancy, the patient underwent a simple mastectomy, and a favorable outcome with low risk of recurrence was anticipated. Postoperative recovery was uneventful, and the patient remained disease-free on follow-up, consistent with the expected benign course of isolated Paget’s disease of the nipple.

Case 7

**Figure 9:**
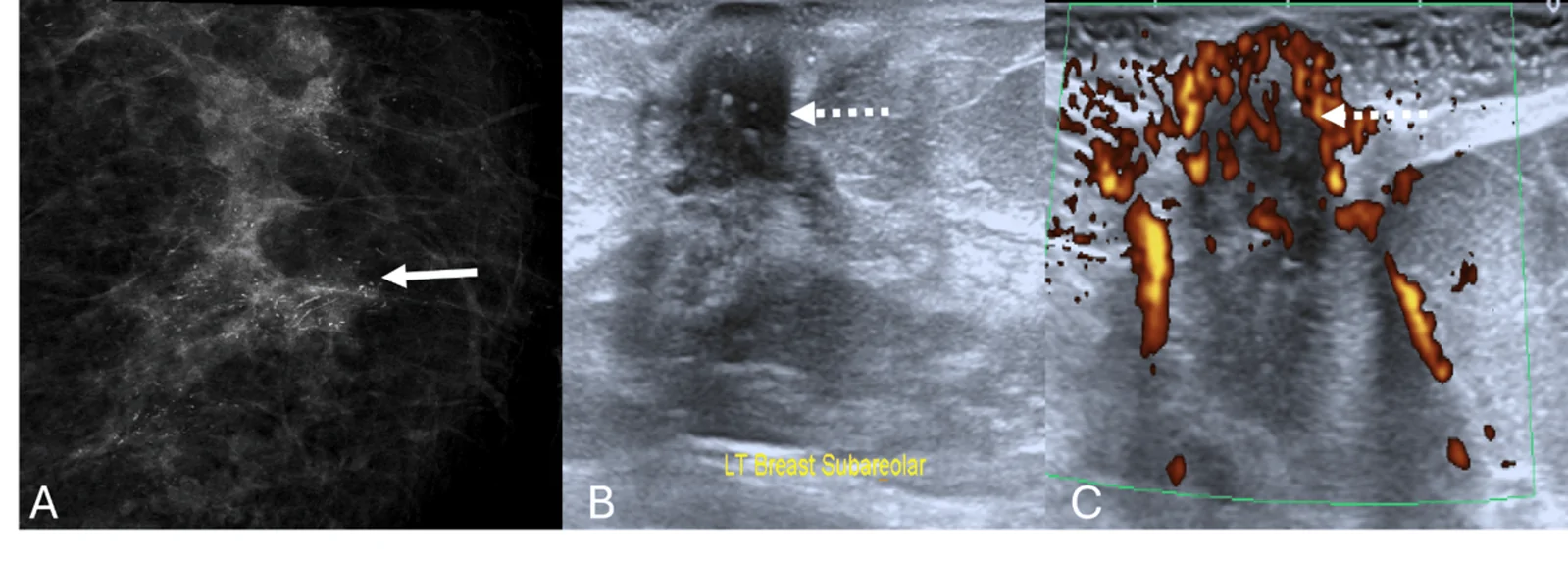
Mammographic and ultrasonographic findings demonstrating pleomorphic calcifications and abnormal nipple-areola complex involvement (A) Spot magnification mammogram of the left breast demonstrating fine linear pleomorphic calcifications in segmental and regional distribution (solid arrow). (B) Ultrasonogram depicting a hetero-echoic left nipple-areolar complex with calcifications and increased vascularity (dotted arrow)

A 43-year-old female patient presented with ulceration of the left nipple of five months’ duration, without a definite palpable lump or nipple discharge. On examination, the left nipple-areola complex showed surface erosion with crusting and mild surrounding skin thickening, and no discrete mass was palpable in either breast; the axillae were clinically unremarkable. Spot magnification mammography of the left breast demonstrated fine linear pleomorphic calcifications in a segmental and regional distribution, raising suspicion for an underlying in situ or invasive process. Ultrasonography depicted a hetero-echoic left nipple-areolar complex with associated calcifications and increased vascularity (Figure 9), and an ultrasound-guided core biopsy was obtained from the retroareolar region. Histopathological examination of the punch and core biopsy specimens confirmed Paget’s disease of the nipple with an underlying DCIS component. Simple mastectomy was planned and was expected to achieve adequate locoregional control; the patient underwent the procedure with an uneventful postoperative course.

Case 8

**Figure 10:**
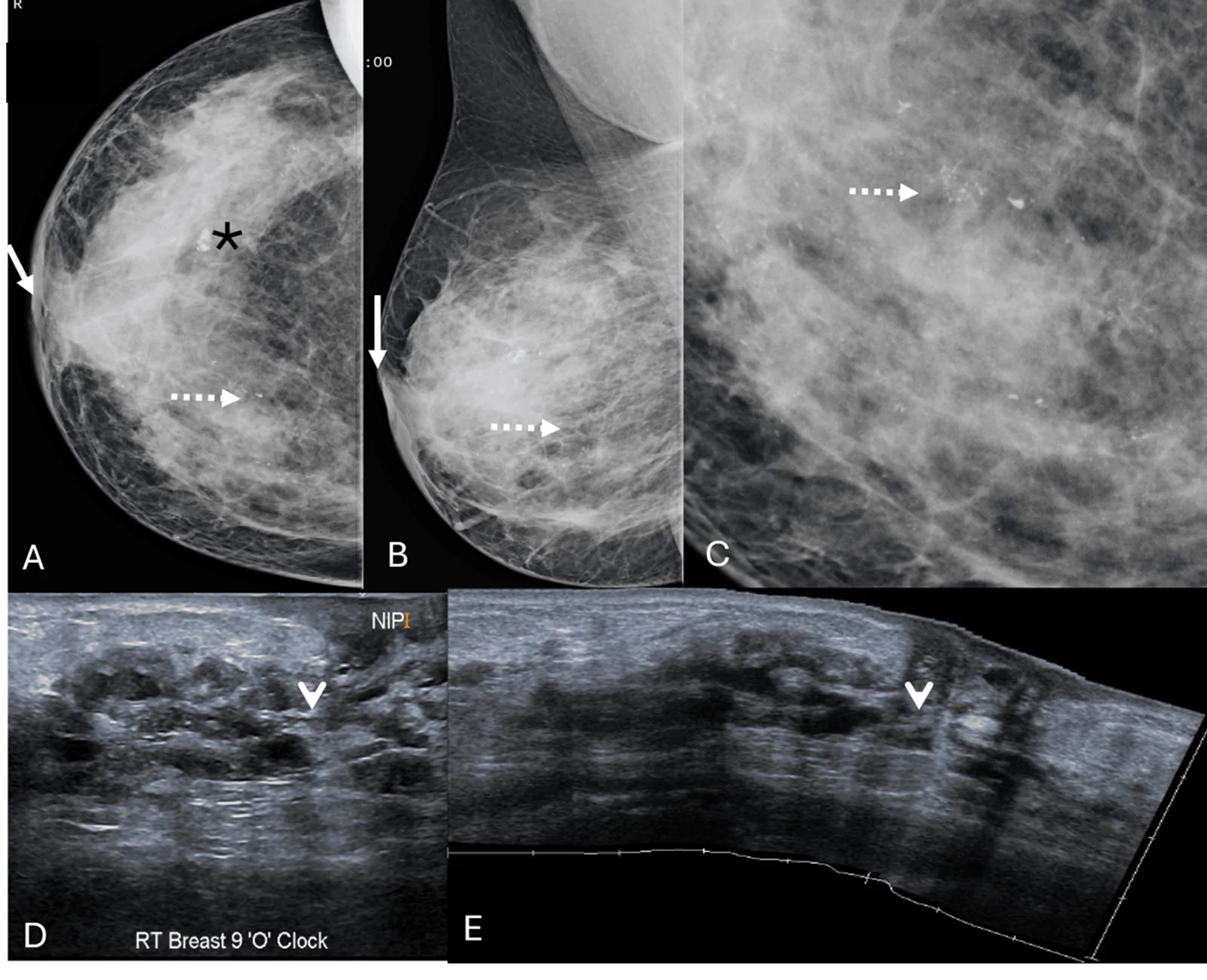
Mammographic and ultrasonographic findings demonstrating retroareolar skin thickening, pleomorphic calcifications, and ductal involvement of the right breast MLO: mediolateral oblique (mammographic view); CC: craniocaudal (mammographic view) (A & B) Mammogram-MLO and CC views of the right breast showing skin thickening in the retroareolar region (solid arrow) and grouped fine pleomorphic calcifications (dotted arrow) in the lower inner quadrant and clustered microcalcifications (asterisk) in the upper outer quadrant. (C) Spot magnification view of the fine pleomorphic calcifications. (D & E) Ultrasonogram representing heterogeneous parenchyma with abnormally dilated ducts showing calcifications (arrowhead) beginning at the nipple

A 47-year-old female presented with right nipple ulceration of eight months' duration. On examination, the right nipple showed surface ulceration with retroareolar skin thickening, and an ill-defined firm area of thickening was palpable in the right breast spanning more than one quadrant; the right axilla was clinically unremarkable, and the left breast was normal. Mammography of the right breast (MLO and CC views) demonstrated skin thickening in the retroareolar region along with grouped fine pleomorphic calcifications in the lower inner quadrant and a separate cluster of microcalcifications in the upper outer quadrant, with spot magnification views confirming their pleomorphic morphology; this distribution of two discrete calcific foci in separate quadrants was considered representative of multicentric disease. Ultrasonography revealed heterogeneous parenchyma with abnormally dilated ducts showing calcifications extending from the nipple (Figure 10), and an ultrasound-guided core needle biopsy was obtained from the dominant area of thickening. Histopathological analysis confirmed Paget’s disease of the nipple with an underlying invasive mammary carcinoma. In view of the patient’s age, multicentric disease distribution, and biopsy-proven invasive carcinoma, modified radical mastectomy was planned as definitive treatment, with the expectation of adequate locoregional clearance. The patient underwent the planned surgery with an uneventful postoperative recovery, consistent with the anticipated outcome. 

A summary of the age, clinical presentation, modalities performed and their principal findings, lesion location, histopathological diagnosis, and surgical management of all eight patients is presented in Table 1.

## Discussion

Clinical presentation

In our cohort, nipple ulceration was the predominant presenting symptom (75%), followed by a palpable breast lump (25%). This is broadly consistent with published data, wherein nipple and areolar changes such as erythema, crusting, excoriation, and ulceration are the most frequently reported initial complaints [5,8]. Approximately 50% of patients with Paget’s disease present with a palpable mass, which is highly indicative of an underlying invasive carcinoma [18]. The relatively lower proportion of patients with a palpable lump in our series may reflect earlier clinical recognition and referral in a tertiary care setting. The presence of a young patient (27 years) with bilateral involvement is noteworthy, as the disease predominantly affects postmenopausal women in their sixth and seventh decades, and bilateral occurrence is exceptionally rare [3,4]. This case underscores the importance of maintaining a high index of clinical suspicion regardless of age.

Mammographic findings

A particularly noteworthy observation in the present study is that all available mammograms demonstrated features suggestive of malignancy, a finding that stands in contrast to prior published series. Kim et al. [19] and Darmiati et al. [20] reported that mammographic findings may be absent or nonspecific in a significant proportion of patients with Paget’s disease. Other series have documented normal mammograms in up to 50% of cases [11,12]. The universally positive mammographic findings in our cohort may be attributable to the use of high-resolution digital mammographic equipment available at a tertiary care institution, advanced radiological expertise, and the fact that the majority of our patients presented with clinically evident disease. This high detection rate should be interpreted with caution, as it reflects a small, highly selected, symptomatic cohort of five patients referred to a tertiary care center, and is subject to significant spectrum and selection bias. It does not represent the general sensitivity of digital mammography in Paget's disease. The mammographic abnormalities identified in our series, including irregular equal-density mass, round circumscribed nodule, fine pleomorphic calcifications, nipple retraction, skin thickening, and axillary lymphadenopathy, are consistent with those described by Sripathi et al. [13] and Sawyer and Asbury [21], who documented a wide spectrum of mammographic presentations ranging from subtle architectural distortions to overt malignant masses. Günhan-Bilgen and Oktay [12], in a series of 52 patients, similarly reported that masses and calcifications were the most common mammographic features, although a notable proportion of their patients had normal or equivocal findings. The discrepancy between our findings and those of earlier studies likely reflects both the evolution of mammographic technology and the referral bias inherent to a tertiary care setting. 

Ultrasonographic findings

Ultrasonography was the most frequently employed imaging modality in our series, performed in 88% of patients, and demonstrated diagnostically relevant findings in all seven patients in whom it was performed. The sonographic findings in our cohort, including irregular hetero-echoic masses, thickened nipple-areola complexes with increased internal vascularity, dilated ducts with calcifications, and axillary lymphadenopathy, are in agreement with those reported by Muttarak et al. [22] and Wei et al. [23]. Muttarak et al. [22], in a review of 16 patients, emphasized the utility of ultrasound in identifying subareolar masses, nipple-areolar complex thickening, and ductal abnormalities, particularly in patients with dense breast parenchyma where mammographic sensitivity is reduced. Wei et al. [23] similarly highlighted the role of sonography in detecting nipple-areolar complex changes and vascularity alterations that may precede or accompany mammographic findings. The demonstration of increased vascularity and posterior acoustic shadowing in some of our cases further supports the value of ultrasound as a complementary tool in the diagnostic workup. Ultrasound is especially relevant in younger patients and those with dense breast tissue, where mammographic sensitivity is inherently limited [13,22]. The identification of axillary lymphadenopathy with cortical thickening in our series is consistent with the known association between Paget’s disease and underlying invasive carcinoma, wherein lymph node metastasis carries significant prognostic implications [3,5]; however, as illustrated by one of our cases, axillary lymphadenopathy may also represent a reactive process in the absence of underlying malignancy, underscoring the need for histopathological confirmation rather than reliance on imaging findings alone.

MRI findings

MRI was performed in 38% of our patients and proved invaluable in delineating the full extent of disease, particularly in identifying features not apparent on mammography or ultrasound. The MRI findings in our cohort included irregular circumscribed enhancing masses with satellite foci, nonmass linear ductal enhancement, nipple erosion, skin ulceration, subcutaneous edema, and diffusion restriction on diffusion-weighted imaging. These findings are strongly corroborated by Lim et al. [11], who described MRI findings in Paget's disease and noted that MRI may identify underlying disease not apparent on mammography or ultrasound. Amano et al. [24] reported a case in which MRI accurately identified underlying DCIS that was mammographically and clinically occult, underscoring the critical role of MRI in patients with normal or equivocal conventional imaging. The nonmass enhancement pattern observed in our cases reflects the intraductal spread characteristic of the DCIS component frequently associated with Paget’s disease, and its accurate delineation on MRI is essential for surgical planning, particularly in determining eligibility for breast-conserving surgery versus mastectomy [11,16,17]. In one patient in our series, MRI identified an irregular mass with satellite foci, a finding consistent with multifocal disease that supported the decision to proceed with mastectomy rather than breast-conserving surgery. The bilateral case in our series demonstrated intensely enhancing nipple-areolar complex involvement on the right and irregular nonmasslike enhancement on the left, a presentation consistent with the findings described by Darmiati et al. [20] in atypical or bilateral cases. MRI-guided surgical planning in such cases is of particular clinical value given the potential for multifocal disease distribution [11,24].

PET-CT findings

PET-CT was performed in 25% of our patients and identified FDG-avid ill-defined lesions with associated skin thickening and lymphadenopathy. Both cases demonstrated significant FDG uptake, consistent with the metabolic activity expected in invasive malignancy. Although PET-CT is not routinely recommended as a first-line modality in the evaluation of Paget’s disease, its utility lies in the detection of locoregional lymph node involvement, distant metastases, and occult multifocal disease [18]. Markarian and Holmes [18] highlighted the evolving role of functional imaging in staging and restaging breast malignancies, noting that PET-CT complements anatomical imaging by providing metabolic characterization of lesions. The findings in our series support the selective use of PET-CT in patients with clinically advanced disease where standard anatomical imaging is insufficient.

Histopathological correlation

Histopathological analysis in our cohort revealed Paget’s disease with underlying invasive mammary carcinoma in 50% of cases, DCIS in 25%, and isolated Paget’s disease of the nipple without underlying breast malignancy in the remaining 25%. The predominance of associated malignancy is consistent with published literature [2,5,18]. The epidermotropic theory, which postulates migration of Paget cells from an underlying DCIS via heregulin-α-mediated motility through the HER2 receptor, accounts for the predominance of associated DCIS or invasive carcinoma in most cases [6]. The isolated Paget’s disease without underlying malignancy observed in our series is consistent with the transformation theory, wherein Paget cells arise from malignant transformation of keratinocytes or Toker cells, a phenomenon reported in fewer than 5% of cases in larger series [7]. The substantially higher proportion observed in our cohort (25%) most likely reflects the small sample size of this series rather than a true difference in disease biology, and should be interpreted with appropriate caution. The IHC confirmation of DCIS using P63 to highlight myoepithelial cells, as demonstrated in one of our cases, exemplifies the importance of ancillary immunohistochemical analysis in establishing the histopathological diagnosis with precision.

Surgical management

The predominance of modified radical mastectomy (63%) in our cohort reflects the advanced stage of disease at presentation and the frequent association with invasive carcinoma or extensive DCIS. Simple mastectomy was performed in 25% of cases, and wide local excision in 12% of cases with less extensive involvement. These findings are broadly consistent with published data, wherein the extent of surgery is determined by the presence, extent, and invasiveness of the underlying malignancy [16,17]. Yao et al. [16] demonstrated that breast-conserving surgery with adjuvant radiotherapy is a viable and oncologically equivalent option in selected patients with Paget’s disease-DCIS or limited invasive disease, provided adequate surgical margins are achieved. Caliskan et al. [17], in a large retrospective series from the European Institute of Oncology, similarly supported breast-conserving therapy as an effective local treatment strategy in appropriately selected patients. The high mastectomy rate in our series likely reflects the advanced or multifocal nature of disease at presentation in a referral center population, rather than a departure from evidence-based surgical principles. Mammographic identification of two discrete foci of calcification in separate quadrants in one patient in our series correctly predicted multicentric disease on histopathology, illustrating the role of mammography in mapping disease extent and guiding the choice between breast-conserving surgery and mastectomy.

Bilateral and atypical presentations

The case of bilateral Paget’s disease in a 27-year-old female patient warrants special discussion. Bilateral Paget’s disease of the breast is exceptionally rare, with only isolated case reports in the literature [4]. Franceschini et al. [4] documented synchronous bilateral Paget’s disease associated with bilateral breast carcinoma, emphasizing the need for comprehensive bilateral evaluation in all patients. The varied and subtle imaging findings in our young bilateral case, including bilateral nipple-areolar thickening on ultrasound, nonmass enhancement on MRI, and calcific specks, are consistent with the diagnostic challenges described by Darmiati et al. [20] and Günhan-Bilgen et al. [12], who noted that atypical or bilateral presentations are particularly prone to diagnostic delay. This underscores the imperative of a structured multimodal imaging workup and heightened clinical suspicion in all suspected cases, irrespective of age or laterality.

Strengths and limitations

The present study is one of the few from South Asia to systematically document the multimodality imaging spectrum of Paget’s disease across all four major imaging modalities: mammography, ultrasonography, MRI, and PET-CT, with histopathological correlation. The inclusion of a rare bilateral case and the availability of complete imaging-pathology correlation in all cases add to the clinical value of this series. However, the study is limited by its retrospective design, small sample size attributable to the rarity of the condition, and single-center recruitment, which may limit generalizability to other demographic groups and healthcare settings. As noted above, the relatively high proportion of isolated Paget’s disease without underlying malignancy observed in this cohort should be interpreted in light of the limited sample size. Future prospective multicenter studies with larger cohorts are warranted to validate these findings and establish standardized multimodality imaging protocols for Paget’s disease of the breast in South Asian populations.

## Conclusions

Paget’s disease of the breast often presents with subtle and insidious clinical and radiological features, which can delay diagnosis. Mammography alone may underestimate disease extent, and a multimodality imaging approach combining ultrasonography, MRI, and, where clinically indicated, PET-CT may improve characterization and assessment of disease extent and support treatment planning. MRI is particularly valuable when conventional imaging is negative or equivocal. PET-CT should be reserved for appropriate staging indications in advanced disease. A high index of clinical suspicion should be maintained in all patients with persistent nipple-areolar changes, regardless of age or laterality. Integrating multimodal imaging techniques enhances the early and precise diagnosis of Paget’s disease of the breast, facilitating personalized treatment strategies and optimizing clinical outcomes.
